# The role of SCF ubiquitin-ligase complex at the beginning of life

**DOI:** 10.1186/s12958-019-0547-y

**Published:** 2019-11-28

**Authors:** Jiayan Xie, Yimei Jin, Guang Wang

**Affiliations:** 1 International Joint Laboratory for Embryonic Development & Prenatal Medicine, Division of Histology and Embryology, Medical College, Jinan University, Guangzhou, 510632 China; 2 School of Public Health, Southern Medical University, Guangzhou, 510515 China; 3 The University of Texas MD Anderson Cancer Center & University of Texas MD Anderson Cancer Center UTHealth Graduate School of Biomedical Sciences, Houston, TX 77054 USA

**Keywords:** SCF, Oogenesis, Spermatogenesis, OET, Embryonic development

## Abstract

As the largest family of E3 ligases, the Skp1-cullin 1-F-box (SCF) E3 ligase complex is comprised of Cullins, Skp1 and F-box proteins. And the SCF E3 ubiquitin ligases play an important role in regulating critical cellular processes, which promote degradation of many cellular proteins, including signal transducers, cell cycle regulators, and transcription factors. We review the biological roles of the SCF ubiquitin-ligase complex in gametogenesis, oocyte-to-embryo transition, embryo development and the regulation for estrogen and progestin. We find that researches about the SCF ubiquitin-ligase complex at the beginning of life are not comprehensive, thus more in-depth researches will promote its eventual clinical application.

As a post-translational modification, ubiquitination controls various cellular processes, such as cell proliferation, cell cycle progression, transcription, and apoptosis. Ubiquitin-proteasome system (UPS) consists of ubiquitin-activating enzyme E1, ubiquitin binding enzyme E2, and ubiquitin protein ligase E3 [1]. Among them, E3 ligase is rather crucial to the determination of specificity of substrates selected by the ubiquitination system. At first, the ubiquitin-activating enzyme E1 adheres to and activates ubiquitin under the condition of ATP energy supply, and then E1 transfers the activated ubiquitin molecule to the ubiquitin-binding enzyme E2. E2 enzyme and various ubiquitin protein ligase E3 aim to recognize substrate proteins and modify them by ubiquitination. The labeled substrate protein is eventually broken down by the proteasome into peptides, amino acids and reusable ubiquitin molecules.

Over 600 speculated E3 ubiquitin ligases encoded in the human genome fall into three major subgroups: the homolog of the E6-AP Carboxyl Terminus(HECT), Plant Homeodomain/U-box (PHD / U-box) and RING finger family [2]. CRL protein family is the most representative kind of ring finger protein E3 ligases, composed of scaffold protein (cullin), bridging protein, substrate receptor protein, and RING protein that recruits E2.CRLproteins contains 8 members [3, 4]: CRL1, CRL2, CRL3, CRL4A, CRL4B, CRL5, CRL7 and CRL9. Among them, Skp1-Cullin-F-box (SCF, also called CRL1) [5, 6] is most typical. The SCF complex consists of s-phase kinase associated protein1 (SKP1), E3 ligase RBX1, Cullin1 (CUL1) and F-box proteins [7, 8].

## The composition and function of SCF complex

The SCF complex is comprised of the unchanging components, including S-phase kinase-associated protein 1 (Skp1), ligase Rbx1 (also known as Roc1), and cullin 1 (Cul1), as well as variable F-box proteins that confer substrate selectivity [5, 6]. The major structural scaffold for the SCF complex is Cul1, which connects the Skp1 domain with the Rbx1 domain. Skp1 is a connexin that binds to Cul1 to form the horseshoe complex and plays a crucial role in identifying and binding F-box. Rbx1 contains a Zinc-binding domain called RING Finger that binds to the E2-ubiquitin conjugate, transferred ubiquitin to the lysine residues of the target protein [9, 10]. As the most crucial component of SCF complex, F-box protein shoulders the responsibility for the recognition of substrates and determines the specificity of the SCF complex.

F-box protein is made up of two main functional domains: various carboxy-terminal domains that bind to specific substrates, and the F-box motif. The F-box motif is a protein-protein interaction domain that was first discovered in F-box only1 (FBXO1; also known as cyclin F) [11] and recruits F-box proteins into the SCF complex via direct binding with the adaptor protein Skp1 [12–14]. First, the F-box protein targets the substrate independently and then binds to the Skp1, so that the substrate is close to the E2 protein to obtain ubiquitin. The F-box protein can regulate the activity of the SCF complexes during the cell cycle. The levels of SCF keep constant during the whole cell cycle, so their activity is determined by the affinity of the F-box protein for the substrate protein. CDK/cyclin-mediated phosphorylation regulates the affinity of this F-box protein. F-box protein family consists of three subclasses, each with different substratum recognition domains. The first one is F-box/WD repeat-containing protein (FBXW) which has WD40 repeat domains. It has a total of ten proteins, including the β-TRCP1, FBXW7 (also called as FBW7 and CDC4) and β-TRCP2 (also called as FBXW11). The second subclass of F-box protein is called leucine-rich repeat protein (FBXL), which contains Sphase Kinase associated Protein 2 (SKP2, also known as FBXL1). F-box only protein (FBXO) with unknown domain is the third subclass, which includes all the unclassified 37 F-box proteins.

Skp1 plays an important role in connecting the catalytic core of the SCF complex to the F-box motif [12, 13, 15]. The Skp1 gene, which is evolutionarily conservative in many species from yeast to humans, encodes the Skp1 protein in humans [16]. The human homologous of the Skp1 gene was first identified in 1993 during the exploration of the cell cycling F using the yeast two-hybrid system [17].Composed of 5 exons, Skp1 encodes 163 amino acids with a molecular weight of 19 kDa [16].The alternative splicing of the gene produces two transcript variants which respectively encode two isotypes: Skp1A and Skp1B. Whether these variants are expressed differentially or positioned in cells has not been precisely defined yet [18].

Cul1 contains three main domains responsible for adjusting its association with other components of the SCF complex. The N-terminal domain of Cul1 mediates its binding to Skp1 [19].It’s C-terminal domain facilitates Cul1 interact with E2 enzymes Cdc34 and E3 enzymes Rbx1/Roc1. The third, also the most conservative region, mediates the adhesion of ubiquitin-like Nedd8 [20]. Nedd8 binds to the 720 arginine residues of Cul1 to strengthen the ubiquitin ligase activity of the SCF complex by increasing its affinity for certain E2 enzymes [21].

## Regulation of SCF complex during gametogenesis and maturation

Life begins with the fertilization of the egg. Thus, the transmission of genes and the formation of life depend largely on the quality of gametes. Any errors in the development and maturation of eggs and sperms can lead to fertilization failure or deficiency of embryonal development, resulting in infertility or miscarriage. Therefore, a complete regulation mechanism of protein degradation is essential for normal meiosis [22, 23]. Therefore, as an important member of UPS, SCF protein complex may be of high significance in life formation.

### The role of the SCF complex in oogenesis

Gametes are generated in primordial germ cells (PGCs). PGCs initially form in the periblast and migrate to the genital ridge. The process of migration is accompanied by continuous division and proliferation. Then morphological changes occur and PGCs become oogonia, which enters the proliferation phase and is surrounded by the granulosa cells of preovulatory follicles to form primordial follicles. This is a very complicated process that requires precise coordination between germ cells and somatic cells and accurate control of genes. Lack of any proteins may hinder germ cells from dividing or dying, so the SCF complex, which regulates proteins, plays a vital role in this process. For example, during the proliferation of drosophila oogonia, Archipelago (AGO) of the F-box protein family mediates the regulation of Cyclin E1 (CCNE1) by the SCF ubiquitin protease system. CCNE1 is a crucial substrate of SCF, which can boost cell transition from G1 phase to S phase by activating cyclin-dependent kinase 2 (Cdk2).The existence and degradation of CCNE1 are strictly regulated by UPS, and its abnormal level can lead to accelerated entry into S phase, causing genetic instability and affecting the time control of mitosis of female germ cells [24]. Therefore, CCNE1 may be used as a target for the detection of female infertility in the future, which deserves further clinical confirmation.

**Fig. 1:**
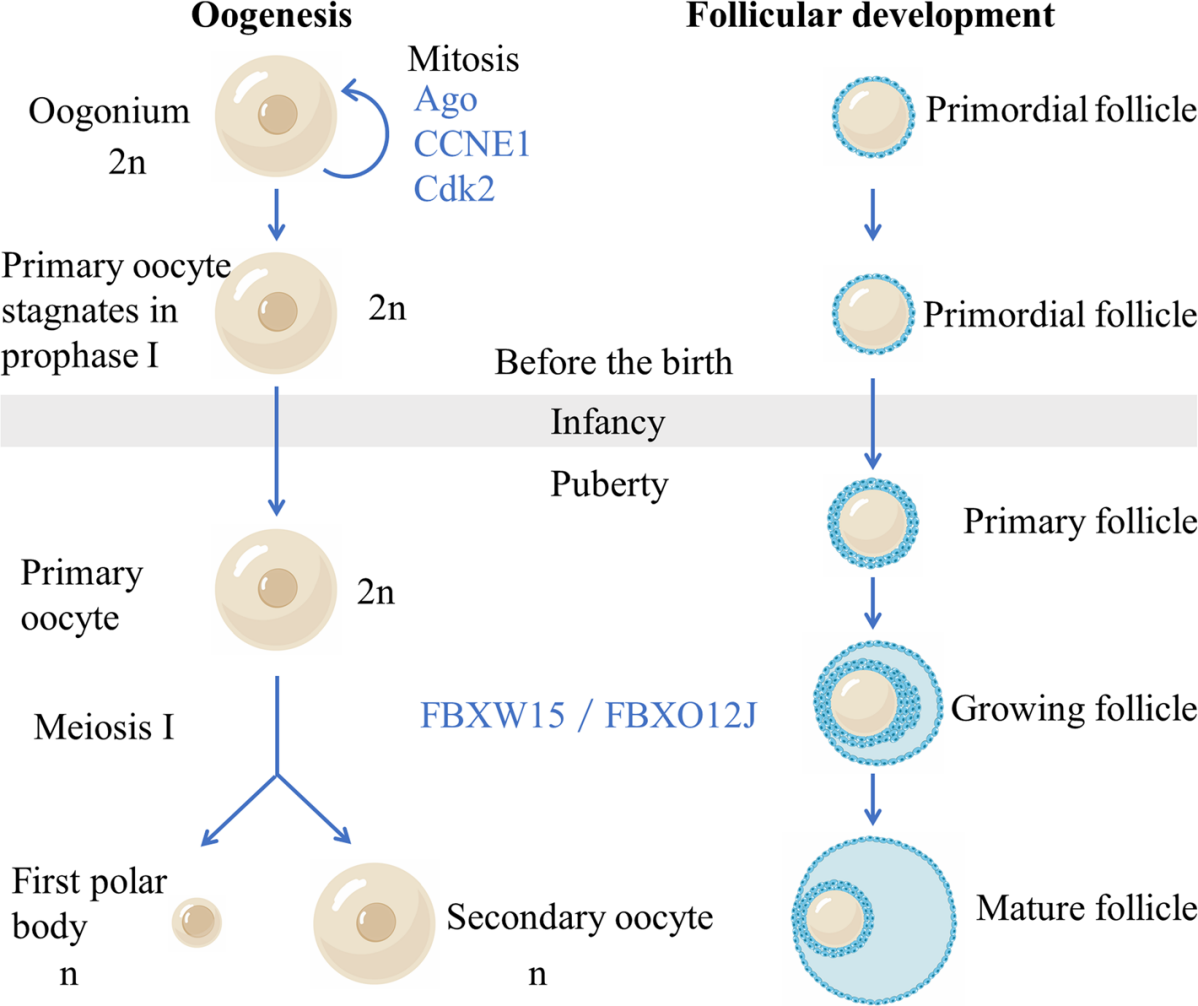
Oogenesis and follicular development. Meiosis occurs, following the mitotic proliferation of oogonia. The oogonia first enters the leptotene. stage of the first meiotic prophase and becomes the primary oocyte. Before birth, the development of primary oocytes stagnates at the zygotene and pachytene stages.Shortlyafter birth, the oocyte enters the diplotene stage. At this moment, the oocyte has a large nucleus, known as germinal vesicle, and gradually forms a primary follicle. Oocytes at this stage are diploid but have four times as much DNA as haploid cells. During this period, the development of oocytes will keep stagnant for a long time, allowing homologous chromosomes to fully perform the transcription of maternal mRNA

Meiosis occurs, following the mitotic proliferation of oogonia (Fig. 1) [25]. The oogonia first enters the leptotene stage of the first meiotic prophase and becomes the primary oocyte. Before birth, the development of primary oocytes stagnates at the zygotene and pachytene stages. At this time, FBXW15 (also known as FBXO12J), a member of the F-box family, which is specifically expressed in ovaries, first appeared in large quantities. Therefore, it is highly possible that FBXW15 / FBXO12J protein prevents the oocyte from reaching the diplotene stage, thus preventing the early end of the first meiotic prophase. Shortly after birth, the oocyte enters the diplotene stage. At this moment the oocyte has a large nucleus, known as germinal vesicle, and gradually forms a primary follicle (Fig. 1). Oocytes at this stage are diploid but have four times as much DNA as haploid cells. During this period, the development of oocytes will keep stagnant for a long time, allowing homologous chromosomes to fully perform the transcription of maternal mRNA. Along with this process, the expression of FBXW15/FBXO12J in the ovary continued to increase after birth, indicating that FBXW15/FBXO12J protein is highly likely to prevent oocytes from further meiosis before ovulation. In conclusion, the fluctuation of FBXW15/FBXO12J expression is very consistent with the timing of early oocyte meiosis and follicular development, suggesting that FBXW15 / FBXO12J protein is highly involved in the regulation of oocytes in different developmental processes. Therefore, previous studies have speculated that FBXW15 / FBXO12J protein is indispensable to fetal and neonatal ovarian development [26]. However, this conjecture has not been confirmed. Does the change of FBXW15 / FBXO12J occur accompanied by the development of oocyte? Or lead to it? Or the other way around? These deserve further discussion.

Though the role of SCF during the development of oocyte is largely unknown, its actual functions and effects are believed to be far greater than what are known.

### The role of SCF complexes in spermatogenesis and development

Spermatogonia stem cells (SSCs) are the origin of sperm. SSCs support spermatogenesis by self-renewal and division. Although some positive regulators of self-renewal have been discovered, little is known about negative regulators. FBXW7 (F-box and WD-40 domain protein 7) is an important negative regulator of SSCs self-renewal. FBXW7 is expressed in an undifferentiated spermatogonium in a cyclin-dependent way. Spermatogonia cell transplantation shows that FBXW7 overexpression reduces SSCs activity, while Fbxw7 deficiency enhances the colonization of SSCs colonization and causes accumulation of undifferentiated spermatogonia, suggesting that Fbxw7 levels are essential for self-renewal and differentiation of SSCs. Further investigations demonstrate that the knockdown of FBXW7 is able to up-regulate myelocytomatosis oncogene (MYC) and CCNE1. FBXW7 negatively regulates the self-renewal of SSCs by degrading MYC [27]. There are also studies that point out that the lack of Peptidyl-prolyl cis/trans isomerase NIMA-interacting 1 (PIN1), which is essential for spermatogenesis, leads to male infertility, while FBXW7 is down-regulated when Pin1 is exhausted [28]. Therefore, FBXW7 could be used as a target to detect male infertility for further clinical verification.

## The role of SCF complex in oocyte-to-embryo transition (OET)

Embryogenesis starts with the fertilization and then triggers series of highly harmonious embryonic development events. This whole transformation process is known as OET [29]. Activation of OET does not need new transcripts, mainly based on the maternal RNA and protein accumulated in fully grown oocytes (FGOs). In this process, the function of nucleus has radical changes: differentiated egg and sperm combine to produce the embryo genome. This change is known as genome reprogramming, a series of epigenetic modifications that transform the genome into a potent state [30].The mechanism and molecular pathway of OET and genome reprogramming are still unknown. Recent studies have compared the transcriptomes of mouse FGOs, two-cell stage murine embryos, Xenopuslaevis FGOs and *Ciona intestinalis* FGOs, to find the transcriptome essential for OET, which is unique to FGOs and highly conserved between species. Members of UPS account for a significant proportion of this transcriptome [31].

After OET activation, maternal materials in the newly fertilized egg control almost all aspects of embryonic development, while the transcription of the zygote genome remains static. After several rounds of rapid cell division in the fertilized egg, maternal mRNA and protein are eliminated and the zygotic genome which controls the early development of life afterward is activated. This process is defined as maternal-to-zygotic transition (MZT) [32]. MZT mainly involves two processes. The first is the clearance of maternal mRNA and protein, which is necessary for oocyte maturation and embryonic development; Then comes the zygotic genome activation (ZGA) [33]. Post translational modifications of various proteins are known to occur during MZT, and ubiquitination is particularly essential. Proteomic analysis showed that SCF complex-associated proteins are highly enriched in mouse fertilized eggs, and many studies have found the specific role of SCF complexes in maternal protein degradation.

Proteomic analysis showed that SCF complex related proteins are highly abundant in mouse fertilized eggs. Many studies have identified specific roles of SCF complexes in maternal protein degradation (Table 1). Precise post-translational regulatory mechanisms, especially SCF-mediated ubiquitination, are crucial for early embryo development.

**Table 1:**
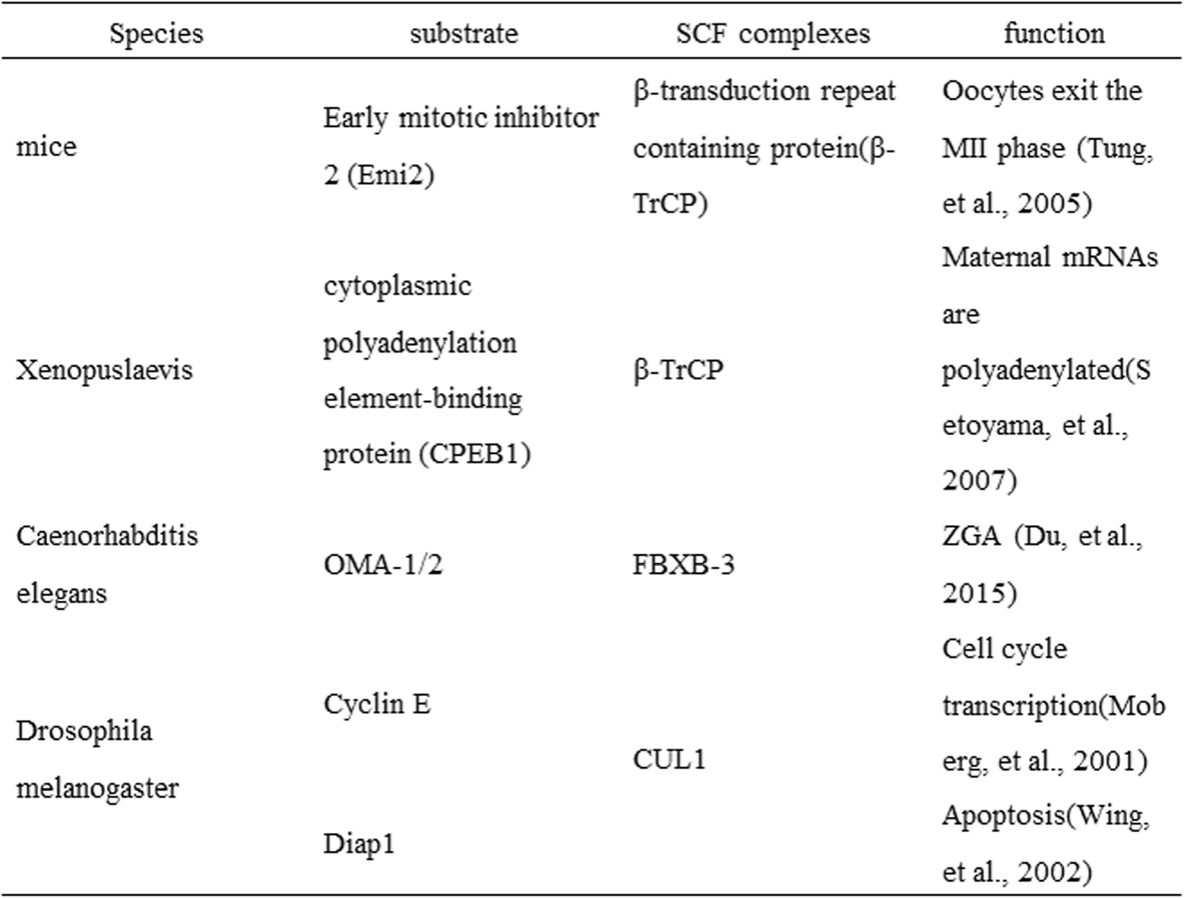
Targeted degradation of maternal proteins in different organisms

The degradation of maternal materials, which is mediated by the SCF complex, is indispensable for embryonic genome activation (EGA). Transcriptomic analysis of bovine embryos at various pre-implantation stages revealed that mRNA of Cul1 and Skp1 were synthesized at early stage of embryo development and activated on day 4 and day 8, suggesting that these transcripts were prepared by the embryo for EGA [34].

Hence, the research lightened us that it is possible to assess infertility related to oogenesis, such as empty follicle syndrome (EFS), by detecting levels of SCF proteins, like Skp1 and Cul1. However, following problems remain to be solved: How does SCF complex affect OET? And how do the errors in this regulation process lead to disease? Scientific researches on these issues are just a start-up. And much more researches are needed to explore the following questions: the relationship of SCF complex with genome reprogramming process during OET, the role of SCF complex in gamete and embryo development, the abnormal levels of SCF complex in diseases, the way how anomaly SCF complex expressions affect the signal pathway, as well as how to apply the results into clinical treatments. Especially for infertility or congenital pediatric diseases, treatment methods are still very limited, and we believe future researches on SCF complex can provide new ideas for new treatments. Although mysteries remain about SCF complex, it is clear that the full control of SCF complex over gamete and embryonic development is essential for the birth and continuation of life.

## The role of the SCF complex for embryonic development and implantation

There are hundreds of different types of cells in our body, arranging from blood cells in vessels to multinucleated myotubes in muscles. For example, nervous system cells including tiny glial cells and meter-long neuron axon, varying widely in morphology and functions. All these cells work together to help the brain deal with complex input signals. Such morphological and functional diversity pervades our entire body. Thus, a developing embryo needs to ensure that over 200 different cells can be differentiated at correct time and place, and precisely regulate them.

The differentiation of cells in embryo requires the specific expression of genes, as well as accurate synthesis and degradation of proteins. And these are precisely controlled by a variety of complex molecular networks of developmental signals. Since subtle differences can cause changes in cell fate, accurate regulation of these signals is a prerequisite for successful differentiation. In recent years, more and more studies have found that protein ubiquitination has become an important regulator of cell fate and function. Abnormal of SCF complex usually leads to birth defects, pediatric diseases or cancer. By forming conjugates of different topologies, ubiquitination can affect the stability, interaction, localization or activity of thousands of proteins, resulting in a wide range of specific signals for cell control [35].

### The role of SCF complex in preimplantation embryo development

The SCF complexes are essential in the development of preimplantation embryos. Studies have found that the development of Cul1^−/−^ embryos are blocked on day 6.5 (E6.5). CCNE1 is highly elevated in all cells of the mutant embryo. For instance, both Cul1^−/−^ blastocyst and trophoblast giant cell have excessive CCNE1 accumulation. The proliferation ability of blastocyst is weakened, while trophoblast giant cells continue to enter the circulation. These findings suggest it is necessary for Cul1 to regulate the protein abundance of CCNE1 to ensure normal embryonic development [36]. What’s more, in preimplantation bovine embryos, the protein level of Cul1 increased gradually from oocyte MII stage to morula stage. And Cul1 mainly locates in the nucleus but a small amount in the cytoplasm. At the blastocyst stage, compared with trophectoderm (TE), the signal in the inner cell mass (ICM) is low. In addition, the level of SKP1 increases remarkably from oocyte MII phase to 4-cellphase, but then decreases sharply. Its localization is similar to that of CUL1 at the blastocyst stage. In the early stage of embryo implantation, activated SCF complexes are evenly distributed throughout the embryo, but TE has more SCF complexes than ICM in the blastocyst stage. All these changes suggest a correlation between SCF complex and preimplantation embryo development [34, 37]. However, the research results are not sufficient to demonstrate a causal relationship between SCF changes and embryo implantation. Moreover, how do embryos precisely regulate signals in time and space to guide cell differentiation? Is there any SCF involved in this? These deserve further exploration.

### The role of SCF complexes in embryo implantation

Human trophoblast progenitor cells differentiatein two different pathways, either to become highly invasive cytotrophoblast cells (CTB) and extravillous trophoblast cells (EVT) or to integrate into syncytio trophoblastic cells [31, 38, 39]. Incomplete trophoblast differentiation can cause poor placental perfusion and even pre-eclampsia (PE). Studies have shown that Cul1 is highly expressed in CTB and EVT in human placenta during early pregnancy. Cul1 siRNA obviously inhibits the growth of villous explants, as well as the invasion and migration of EVT-derived HTR8/SVneo cells. This inhibition also results in decreased lytic activity of matrix metalloproteinase 9 (MMP-9) and increased expression of MMP inhibitors in tissues (TIMP-1 and -2). On the contrary, exogenous Cul1 proteins continue to promote invasion and migration of HTR8/SVneo cells. Obviously, during trophoblastic cell fusion, Cul1 protein gradually reduces, while Cul1 siRNA largely strengthens the integration of BeWo cells induced by forskolin. The level of Cul1 protein in placental villi of the control group is significantly higher than that of PE. Namely, Cul1 promotes the invasion of human trophoblast cells, and Cul1 expression disorder may be related to PE [40]. Therefore, further studies on Cul1 levels in early pregnancy are likely to provide new ideas for the diagnosis of PE.

### The role of SCF complexes in the development of embryonic organ system

Many cells in embryos undergo epithelial-mesenchymal transition (EMT) at least once before terminal differentiation, and this process is also regulated by SCF complexes. Typically, except for the neural crest development, EMT includes the invasion of the mesoderm, the formation of the heart valve and the development of the secondary palate, etc. [41–43]. The central transcription factors that regulate developmental EMT include SMAD interacting protein 1 (Sip 1), Snail, Twist and Snail protein homolog (Slug). These factors play a role in embryonic precursor cell formation and subsequent EMT migration [44, 45]. Also, these EMT regulators are regulated by ubiquitination by Ppa from the F-box family [46]. For example, the EMT regulator Snail protein is mainly regulated by Ppa protein-mediated UPS in embryos [47].. Additionally, the vertebrate F-box/wd40-repeat protein (β-TrCP), also belonging to the F-box family, is able to ubiquitinate IκBα proteins. After that, the ubiquitinated IкBα protein activates the nuclear factor кB (NF-кB) to enter the nucleus and activate its target genes Twist and Snail. Furthermore, after knocking out the F-box domain of the Slimb protein in Drosophila embryos, the IкBα protein cannot be ubiquitinated, and subsequent NF-кB protein-mediated transcription is also inhibited. At the same time, Twist and Snail cannot be activated normally [48]. These experimental results indicate that SCF proteins function as a significant regulator in development-related EMT processes. Defects in these proteins not only cause ubiquitination abnormalities, but also affect development-related EMT. It causes a series of congenital developmental defects such as neural crest dysplasia, heart valve defects, secondary palate defects, etc.

**Fig. 2:**
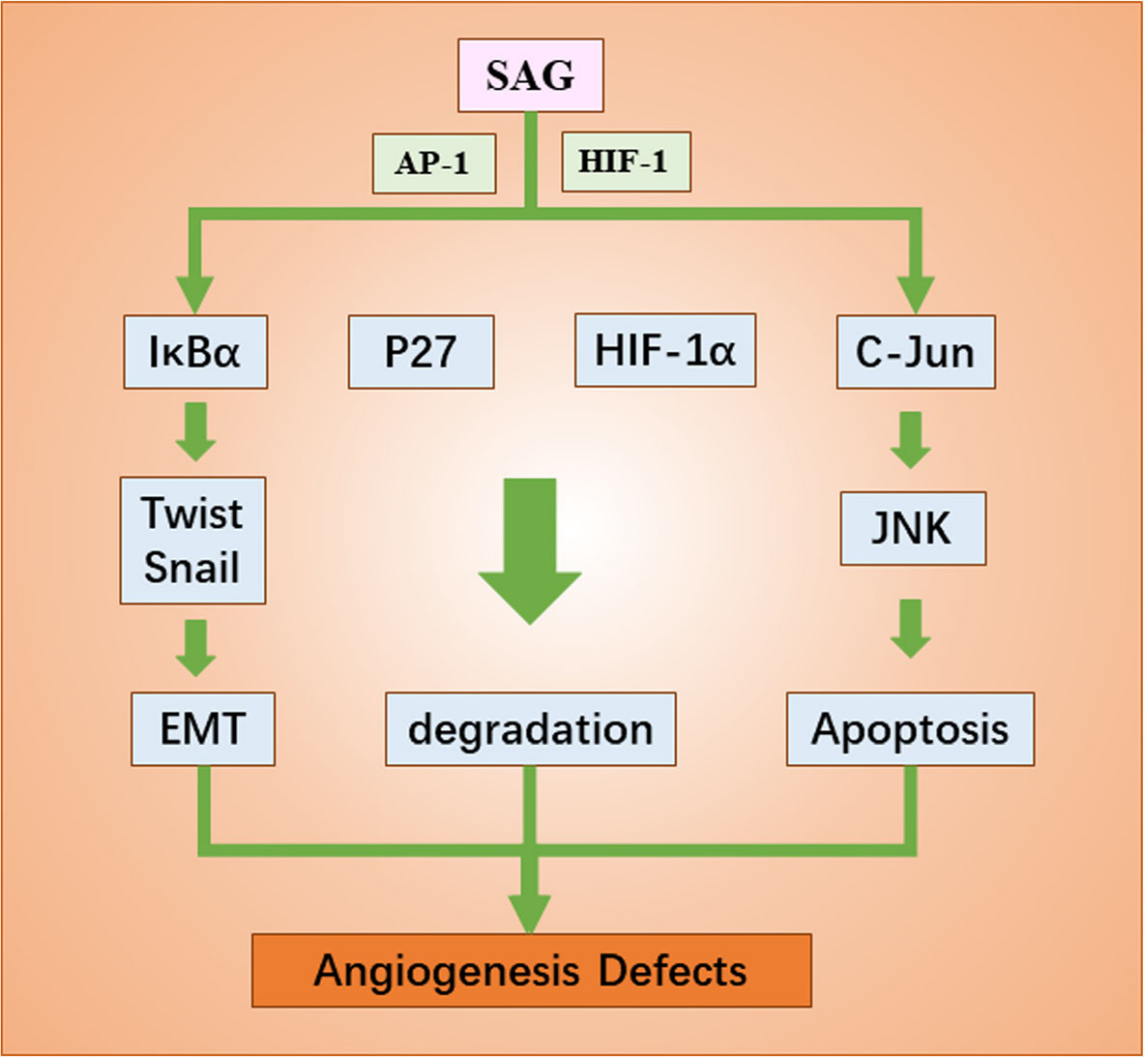
The role of SAG in the development of embryonic organ system. Under the stimulation of ROS, mitogen and hypoxia environment, SAG transcription was induced by AP-1and HIF-1. SAG proteins can recruit other components of CRL to boost ubiquitination and degrade various substrates, including P27, C-Jun, IκBα and HIF-1α, etc. Without SAG, those proteins cannot be ubiquitinated and affect the process of EMT, degradation and apoptosis, causing angiogenesis defects and even embryonic death

Embryonic development is accompanied by cell proliferation and mitosis. Previous studies find that AGO affects mitosis through ubiquitination and degradation of cell cycle and cell growth related proteins. AGO not only inhibits the proliferation of Drosophila cells, blocks tumorigenesis in mammals, but also works in embryonic organogenesis. AGO functions in forming tracheal systems in drosophila embryos through the Trachealess (Trh), a conservative Basic-helix-loop-helix-PAS (bHLH-PAS). The ubiquitin ligase AGO can affect the development of tracheal system of drosophila embryo by controlling Trh protein and its substrate Breathless protein [49]. The role of AGO protein in other species is also related to organogenesis. For example, in mice, FBXW7 (the homologous of AGO) is essential for the normal development of cardiovascular system [50]. In addition to AGO, sensitive to apoptosis gene (SAG, also known as RBX2 or ROC2), a RING protein of the SCF family, also plays a significant role in embryogenesis [3, 11, 51]. SAG proteins can recruit other components of CRL to boost ubiquitination and degrade various substrates, including P27 [52], C-Jun [53], Pro-caspase-3 [54], IκBα [55], HIF-1α [56], NOXA and NF-1, etc. The absence of SAG leads to excessive P27 protein, causing angiogenesis defects and embryonic death [57] (Fig. 2). In conclusion, ubiquitination abnormality and accumulation of substrates caused by SCF protein deletion can cause organogenesis defects and even embryonic death. This again highlights the importance of SCF in the process of embryonic organogenesis. But how to put these findings to clinical use? These all remain to be explored.

## F-box protein family and estrogen and progesterone

Oocyte development, ovulation and periodic changes in endometrium are closely regulated by estrogen and progesterone. While estrogen and progesterone work along with the F-box protein in some physiological processes. Among them, 17α-ethinylestradiol (EE_2_) can affect the expression of F-box protein by various ways, decreasing the expression of Fbxl14a, Fbxl14b, Fbxo25 and β-TRCP2b, and increasing the expression of S phase kinase associated protein 2 (Skp2) [58]. Other studies have shown that SCF-Skp2/Cks1 can regulate P27, cyclin-dependent kinase inhibitor, to affect the endometrium according to the levels of estrogen and progesterone. Under the induction of estrogen, P27 is phosphorylated, which is then ubiquitinated and degraded by SCF-Skp2/Cks1.Estrogen can also result in the degradation of P27 protein by maintaining the integrity of Skp2 and Cks1. Estrogen promotes endometrial hyperplasia through these two mechanisms, while progesterone has opposite effects on P27, Skp2 and Cks1, thereby inhibiting endometrial hyperplasia. Therefore, F-box proteins exert an important impact on regulating menstrual cycle. In addition, it has been reported that Skp2-mediated degradation of P27 is the main molecular mechanism of estrogen-induced endometrial carcinogenesis (EC). Therefore, preventing Skp2/Cks1-mediated degradation of P27 or reducing the level of Skp2-Cks1 maybe a new way to prevent and treat type I EC [59].

## Conclusion

As is known to all, UPS - mediated ubiquitination is an important pathway for post-translational protein modification. SCF complex, a core member of UPS, plays an important role in nearly all aspects of human reproduction. SCF is involved in the ubiquitination of key proteins in cell cycle, cell proliferation and differentiation, EMT, cell signal transduction, etc. In order that, it can participate in the maturation of gametes, OET, embryonic development, and can also function together with estrogen and progesterone in vivo. In the era of genome editing, we should combine biochemical mechanism with clinical researches to further analyze the role of ubiquitination in human reproductive and development-related diseases, in order to provide new insights for the early diagnosis and treatment of infertility and maternal diseases. We hope that core members of SCF can serve as potential targets for unsolved problems during human gametogenesis, fertilization, early embryo implantation and development, as well as new assisted reproductive technologies such as cloning and oocyte cytoplasmic donation.

## Data Availability

All data supporting the conclusion of this article are included in this published article.

## Abbreviations

AGO: Archipelago
bHLH-PAS: Basic-helix-loop-helix-PAS
CCNE1: Cyclin E1
Cdk2: Cyclin-dependent kinase 2
CTB: Cytotrophoblast
EC: Endometrial carcinogenesis
EE2: 17α-ethinylestradiol
EFS: Empty follicle syndrome
EGA: Embryonic genome activation
EMT: Epithelial-mesenchymal transition
EVT: Extravillous trophoblast
FBXO: F-box only protein
FBXO1: F-box only1
FBXW7: F-box and WD-40 domain protein 7
FGOs: Fully grown oocytes
HECT: The homolog of the E6-AP Carboxyl Terminus
ICM: Inner cell mass
MMP-9: Matrix metalloproteinase 9
MYC: Myelocytomatosis oncogene
MZT: Maternal-to-zygotic transition
NF-кB: The nuclear factor кB
OET: Oocyte-to-embryo transition
PE: Pre-eclampsia
PGCs: Primordial germ cells
PHD: Plant Homeodomain
PIN1: Peptidyl-prolyl cis/trans isomerase NIMA-interacting 1
SCF: Skp1-Cullin-F-box
Sip1: Smad interacting protein 1
SKP1: S-phase kinase associated protein1
SKP2: Sphase Kinase associated Protein 2
Slug: Snail protein homolog
SSCs: Spermatogonia stem cells
TE: Trophectoderm
Trh: Trachealess
UPS: Ubiquitin proteasome system
ZGA: Zygotic genome activation
